# Dietary Potassium Supplementation Reduces Chronic Kidney Lesions Independent of Blood Pressure in Deoxycorticosterone-Acetate and High Sodium Chloride-Treated Mice

**DOI:** 10.3390/ijms242316858

**Published:** 2023-11-28

**Authors:** Qing Wang, Stephan C. Schäfer, Jacques-Antoine Haefliger, Marc P. Maillard, Florian Alonso

**Affiliations:** 1 Division of Nephrology and Hypertension, Lausanne University Hospital (CHUV), 1011 Lausanne, Switzerland; marc.maillard@chuv.ch; 2 Institute for Pathology, Uniklink Köln, Kerpener Strasse 62, 50937 Köln, Germany; stephan.schaefer@pathologie-fn.de; 3 Department of Biomedical Sciences, Faculty of Biology and Medicine, Lausanne University, Bugnon 7a, 1005 Lausanne, Switzerland; jacques-antoine.haefliger@chuv.ch; 4 BioTis, Université de Bordeaux, INSERM U1026, 146 Rue Léo Saignat, 33076 Bordeaux, Cedex, France; florian.alonso@u-bordeaux.fr

**Keywords:** potassium, sodium, deoxycorticosterone-acetate, inflammation, fibrosis, gene, mice

## Abstract

We have previously shown that an excess of deoxycorticosterone acetate and high sodium chloride intake (DOCA/salt) in one-renin gene mice induces a high urinary Na/K ratio, hypokalemia, and cardiac and renal hypertrophy in the absence of hypertension. Dietary potassium supplementation prevents DOCA/salt-induced pathological processes. In the present study, we further study whether DOCA/salt-treated mice progressively develop chronic inflammation and fibrosis in the kidney and whether dietary potassium supplementation can reduce the DOCA/salt-induced renal pathological process. Results showed that (1) long-term DOCA/salt-treated one-renin gene mice developed severe kidney injuries including tubular/vascular hypertrophy, mesangial/interstitial/perivascular fibrosis, inflammation (lymphocyte’s immigration), proteinuria, and high serum creatinine in the absence of hypertension; (2) there were over-expressed mRNAs of plasminogen activator inhibitor-1 (PAI-1), fibronectin, collagen type I and III, interferon-inducible protein-10 (IP-10), monocyte chemotactic protein-1 (MCP1), transforming growth factor-*β* (TGF-*β*), tumor necrosis factor-alpha (TNF-*α*), osteopontin, Nuclear factor kappa B (NF-κB)/P65, and intercellular adhesion molecule (ICAM)-1; and (3) dietary potassium supplementation normalized urinary Na/K ratio, hypokalemia, proteinuria, and serum creatinine, reduced renal hypertrophy, inflammations, and fibrosis, and down-regulated mRNA expression of fibronectin, Col-I and III, TGF-*β*, TNF-*α*, osteopontin, and ICAM without changes in the blood pressure. The results provide new evidence that potassium and sodium may modulate proinflammatory and fibrotic genes, leading to chronic renal lesions independent of blood pressure.

## 1. Introduction

Studies have documented that aldosterone regulates sodium and potassium homeostasis. Aldosterone and mineralocorticoids activate the mineralocorticoid receptor (MR) causing inflammation and fibrosis in the heart, fibrosis, remodeling in the blood vessels, tubulointerstitial fibrosis and glomerular injury in the kidney, etc. However, all the studies have consistently demonstrated that concomitant high sodium intake and hypertension are obligatory components of this process [1,2,3,4,5,6]. We have shown that an excess of mineralocorticoid alone has no harmful effects on the heart in one-renin gene normotensive mice [7]. We have also demonstrated that among different pathogenic factors such as hypertension, mineralocorticoid excess, potassium depletion, and high sodium intake, the latter is critical for the development of renal and cardiac hypertrophy, cardiac perivascular fibrosis, and left ventricular dysfunction in one-renin gene DOCA/salt normotensive mice [7]. Moreover, dietary potassium supplementation can prevent DOCA/salt-induced renal and cardiac hypertrophy [8]. In the present study, we investigate (1) whether long-term DOCA/salt administration induces a chronic and progressive renal injury, including inflammation, fibrosis, and proinflammatory and fibrotic gene upregulation in the kidney in one-renin normotensive mice; and (2) whether dietary potassium supplementation reduces the DOCA/salt-induced renal injury and down-regulates proinflammatory and fibrotic gene expressions in the kidney in these mice.

## 2. Results

### 2.1. DOCA/Salt-Induced Renal Lesion and Proinflammatory and Fibrotic Marker Genes in the Absence of Hypertension

**Figure 1:**
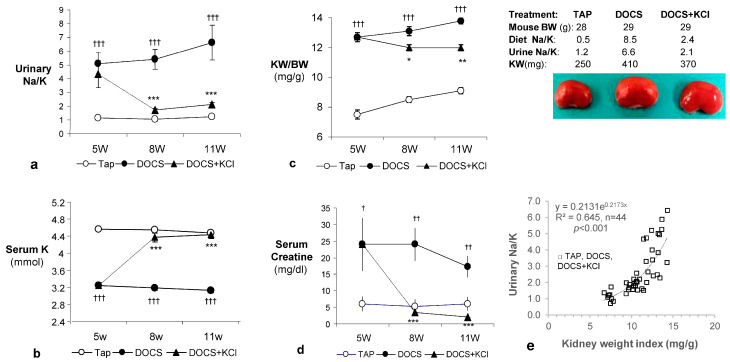
Late dietary potassium supplementation decreases urinary Na/K ratio (**a**), corrects hypokalemia (**b**), partially reverses renal hypertrophy (**c**), and recovers renal dysfunction (**d**) in DOCS mice. (**e**) A significant correlation exists between urinary Na/K ratio and kidney weight index among TAP, DOCS, and DOCS + KCl-treated mice. Data are mean ± SEM, * *p* < 0.05, ** *p* < 0.01, *** *p* < 0.001 DOCS vs. DOCS + KCl; ^†^ *p* < 0.05, ^††^ *p* < 0.01, ^†††^ *p* < 0.001 DOCS vs. TAP control, *n* = 9–12 per group.

Blood pressure (BP), heart rate (HR), serum Na^+^, and body weight were comparable at the end of weeks 5, 8, and 11 between DOCA/salt (DOCS) and Tap water (TAP)-treated mice (Table 1). However, the mice were kept on a high sodium intake with 1% NaCl fluid, followed by a high urinary Na^+^ excretion expressed as U_Na/Creat_ (Table 1) and a high urinary Na/K ratio (Figure 1a). Serum K^+^ level (mmol/L) in DOCS mice was significantly lower (hypokalemia: serum K < 3.5 mmol) than those in TAP-treated animals (Figure 1b). Kidney weight (KW) and KW/body weight (BW) ratio (kidney weight index: determination of renal hypertrophy) in DOCS mice were significantly increased compared to those in the TAP group (Table 1 and Figure 1c). In addition, there was a significant correlation between urinary Na/K ratio and kidney weight index among TAP, DOCS, and DOCS + KCl treated mice (Figure 1e).

**Figure 2:**
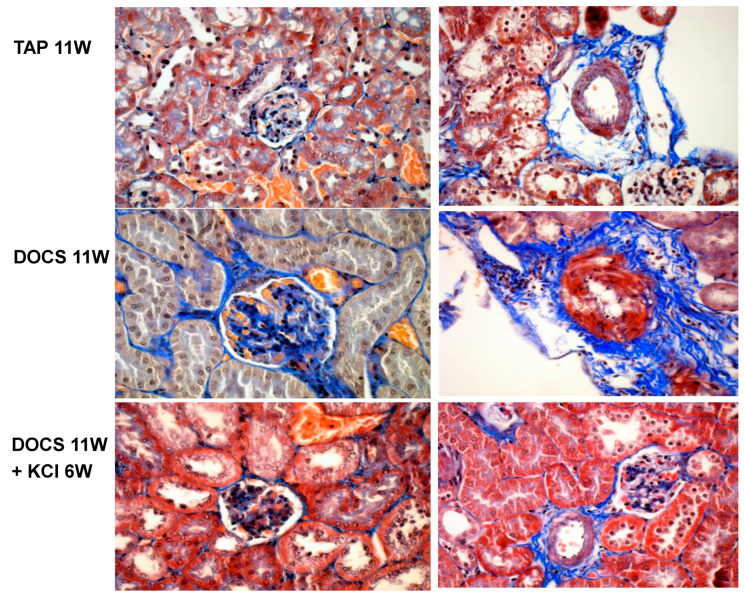
Representative examples of renal morphology (original magnification 40×, under oil immersion). The left pictures are glomerulus and tubules; the right are renal arterioles. Histological examinations revealed extensive mesangial and perivascular fibrosis (Mason staining, blue color), lymphocytes around the hypertrophic vessel, and tubular hypertrophy in 11-week DOCS-treated mice compared to the TAP control group. Even late dietary potassium supplementation for 6 weeks can reverse the renal injury in DOCS mice. W: week.

**Figure 3:**
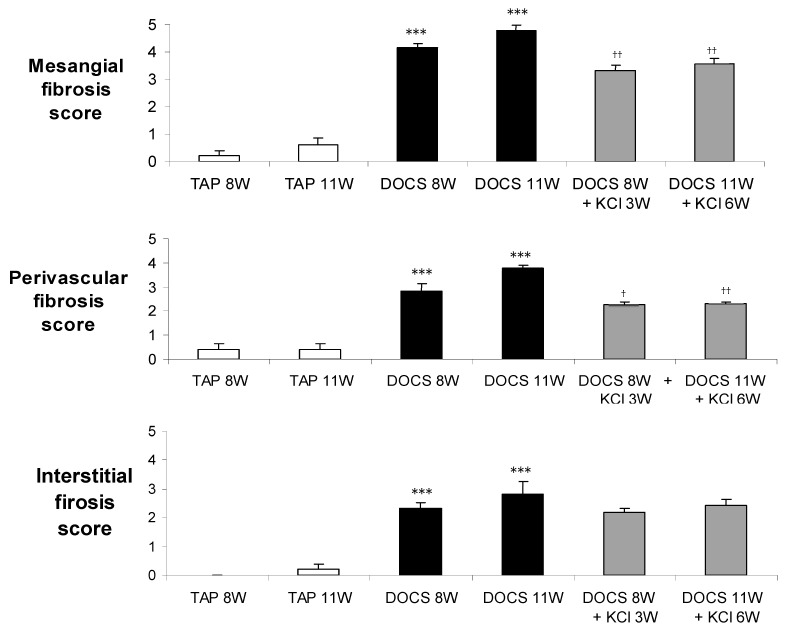
Semiquantitative analysis of renal fibrotic lesions. *** *p* < 0.001 DOCS vs. TAP control, ^†^ *p* < 0.05 and ^††^ *p* < 0.01 DOCS vs. DOCS+KCl. *n* = 5–7 per group. W: week.

**Figure 4:**
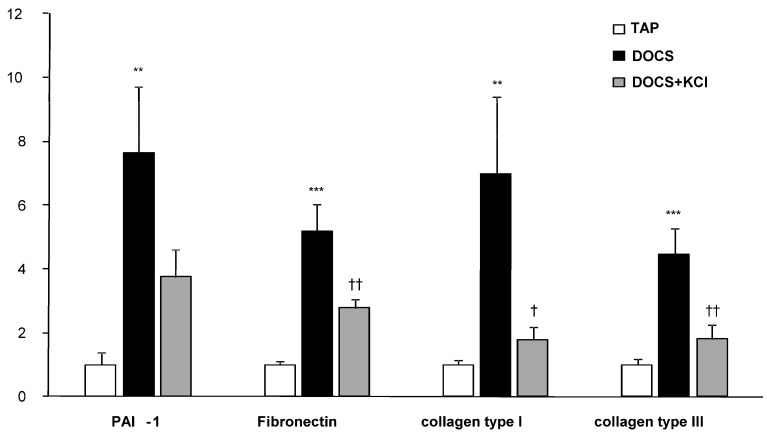
Fibrosis-related gene expression (mRNA) in mice kidney. PAI-1: plasminogen activator inhibitor-1. *** *p* < 0.001, ** *p* < 0.01 DOCS vs. TAP, ^††^ *p* < 0.01, and ^†^ *p* < 0.05 DOCS + KCl vs. DOCS. *n* = 7–9 per group.

**Figure 5:**
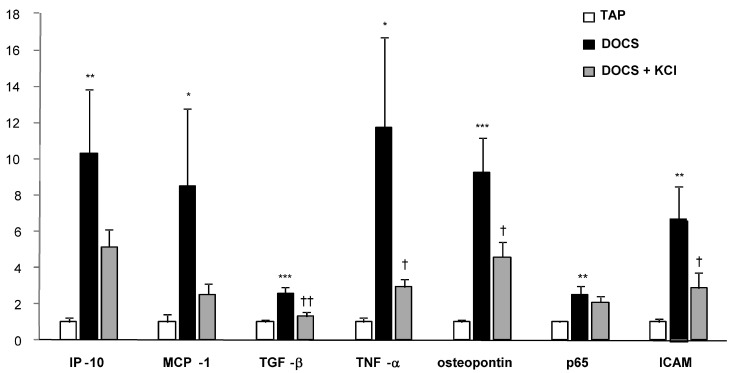
Inflammation-related gene expression (mRNA) in mice kidney. IP-10: interferon-inducible protein-10; MCP1: monocyte chemotactic protein-1; P65: Nuclear factor-kappaB (NF-kB); ICAM intercellular adhesion molecule-1, CD54. *** *p* < 0.001, ** *p* < 0.01, * *p* <0.05 DOCS vs. TAP, ^††^ *p* < 0.01, and ^†^ *p* < 0.05 DOCS+KCl vs. DOCS. *n* = 7–9 per group.

After 8 weeks of DOCS treatment, mice developed tubular/vascular hypertrophy, mesangial, interstitial, and perivascular fibrosis, and lymphocyte immigration in the kidney compared with TAP control animals (Table 2, Figure 2 and Figure 3). Urinary protein to creatinine ratios (U_Prot/Creat_) in DOCS mice were higher (*p* < 0.01) than those in the TAP group (Table 1) over the experimental period. Serum creatinine levels (mg/dL) in DOCS mice in weeks 8 and 11 were significantly higher (*p* < 0.001) than those in TAP control mice (Figure 1d), indicating that DOCS mice developed renal dysfunction or failure. In addition, in the kidneys of DOCS mice, there was a significant increase in mRNA expression of plasminogen activator inhibitor-1 (PAI-1), fibronectin, collagen type I and III interferon gamma-induced protein-10 (IP-10), monocyte chemoattractant protein-1 (MCP-1), transforming growth factor-*β* (TGF-*β*), tumor necrosis factor-alpha (TNF-*α*), osteopontin, p65, and intercellular adhesion molecule (ICAM) in comparison with TAP control animals (Figure 4 and Figure 5).

### 2.2. Dietary Potassium Supplementation Reduces Renal Lesion and Proinflammatory and Fibrotic Marker Gene Expressions in DOCA/Salt Normotensive Mice

After dietary potassium supplementation in DOCS mice for 6 weeks, urinary K^+^ excretion (U_K/Creat_) increased 2- to 3-fold in DOCS+KCl mice compared to TAP and DOCS animals (*p* < 0.001; Table 1). Urinary Na^+^ excretion (U_Na/Creat_) was comparable between the DOCS and DOCS+KCl groups (Table 1). Consequently, dietary potassium supplementation induced a 2- to 3-fold decrease in urinary Na/K ratio in DOCS+KCl mice compared to the DOCS group (Figure 1a).

In addition, in DOCS+KCl mice, dietary K^+^ supplementation raised serum K^+^ (mmol/L) to the same level as in TAP control animals (Figure 1b). K^+^ supplementation significantly reduced the KW/BW ratio (mg/g) in the DOCS+KCl group compared to the DOCS group (Figure 1c). K^+^ supplementation significantly reduced interstitial, mesangial, perivascular fibrosis, lymphocyte infiltration (Figure 2 and Figure 3) in the kidney, and proteinuria (Table 1) in DOCS+KCl mice compared to DOCS animals. The high serum creatinine in DOCS mice was completely reversed by dietary K^+^ supplementation (Figure 1d), whereas BP was not altered during K^+^ supplementation (Table 1), suggesting that all changes caused by dietary K^+^ supplementation are BP-independent. Dietary K^+^ supplementation also reduced or normalized the renal mRNA expressions of TGF-β, TNF-α, osteopontin, ICAM, fibronectin, and Col-I and III in DOCS in DOCS+KCl mice compared to DOCS animals (Figure 4 and Figure 5).

## 3. Discussion

In the present study, we observed that after administration of DOCA and a high sodium diet (DOCS) to uni-nephrectomized one-renin gene mice for 11 weeks, the DOCS mice developed extensively tubular and vascular hypertrophy (Table 2), mesangial, interstitial, and perivascular fibrosis with lymphocyte infiltration in the kidney, indicating kidney chronic inflammation (Figure 2 and Figure 3, Table 2). The DOCS mice also developed a high urinary Na/K ratio (≥5, Figure 1a), albuminuria (or proteinuria, Table 1) over the experimental period, and high serum creatinine (indicating renal dysfunction, Figure 1d). However, the one-renin gene DOCS mice maintained normal blood pressure (Table 1) over the experimental period. More interestingly, dietary K^+^ supplementation in DOCS mice (DOCS+KCl group) for 6 weeks normalized urinary Na/K ratio (Figure 1a), serum potassium and creatinine levels (Figure 1b,d), and partially but significantly reduced renal hypertrophy, inflammation, and fibrosis (Figure 1, Figure 2 and Figure 3, Table 2). Although mechanisms of DOCS-induced damage to target organs (heart, kidney, vessels, etc.) remain largely unsolved, several mediators may be concerted for the development of renal lesions in the DOCS one-renin gene normotensive mouse model, such as high sodium intake, potassium depletion or hypokalemia, mineralocorticoids excess, intrarenal ischemia, vascular endothelial dysfunction, etc.

It is well known that hypokalemia is associated with renal hypertrophy and tubulointerstitial disease in both experimental animals and humans [9,10,11,12]. Previously, we demonstrated that DOCS-induced cardiac and renal hypertrophy was associated with hypokalemia [8]. In DOCS one-renin gene normotensive mice with severe hypokalemia (serum K: 2.76 ± 0.14), we have observed cardiac and vascular hypertrophy, left ventricular dysfunction, and coronary perivascular fibrosis with upregulation of cardiac Col-I and III fibrotic genes as well as overproduction of their proteins. The mice on a low sodium diet did not develop cardiac hypertrophy, fibrosis, and dysfunction with the same amount of DOCA administration [7], suggesting that high sodium intake is a critical factor for target organ damage. Consequently, in the present study, we further observed that long-term DOCS-treated one-renin gene mice developed chronic renal inflammation, fibrosis, and dysfunction without hypertension (Figure 1d and Figure 2, Table 1, Figure 4 and Figure 5). Recent studies have shown that DOCS not only activates epithelial sodium channels (ENaC) in the kidney to generate hypervolemia hypertension and circulatory system remodeling but also activates endothelial sodium channels (EnNaC). The presence of both MR and EnNaC activation by aldosterone/mineralocorticoids and high salt intake contributes to vascular flow-mediated dilation, nitric oxide-mediated vasorelaxation, arterial stiffening, etc. [13,14,15,16]. Previously, using the dorsal skin fold chamber model for intravital microscopy, we have examined endothelium-dependent vasorelaxation of precapillary resistance arterioles in response to acetylcholine or the NO donor SNAP in awake mice. In DOCS one-renin gene normotensive mice with ENaC activation (high amiloride-sensitive rectal potential difference) and EnNaC activation as well [7], the relaxation of resistance arterioles was blunted in response to acetylcholine and, to a lesser extent, to SNAP, indicating endothelium-dependent vasorelaxation dysfunction. This endothelium dysfunction can be restored with dietary K^+^ supplementation [17]. In the present study, as shown in the Figure 2 DOCS 11W intrarenal arterioles image and in Table 2, there was a significant increase in vascular wall thickness with a relatively smaller lumen that may induce ischemia, endothelium-dependent vasorelaxation dysfunction, and trigger inflammation and fibrosis in the kidney.

Proinflammatory and immunomodulatory cytokines, such as IL-1, -6, and -8, TGF-*β*, TNF-α, and MCP-1 (monocyte chemoattractant protein-1), can be produced by leukocytes and released into the renal tissue and the circulation. These cytokines, induced in a setting of immune cell activation, may cause endothelial and vascular dysfunction, cardiac, renal, and neurological damage, and increased blood pressure [18]. Various cell types secrete IP-10 in response to IFN-γ. They include monocytes, endothelial cells, and fibroblasts. TNF-α is the best-known proinflammatory cytokine and is released by many cell types, including macrophages, neutrophils, and dendritic cells. It has also been identified as one of the cytokines that mediate end-organ damage in models of hypertension associated with inflammation. Its production is in response to various stimuli, including LPS, Ang II, and high salt intake, and the local overproduction of TNF-α may decrease NaCl reabsorption, extracellular fluid volume, and blood pressure [19,20,21,22]. TGF-*β*, NF-kB, ICAM, and fibronectin were documented to mediate renal inflammation and fibrosis. Osteopontin is a matricellular protein that mediates cell migration, adhesion, and survival in many cell types and mediates the pathogenesis of a variety of disease states, including atherosclerosis, glomerulonephritis, cancer, and chronic inflammatory diseases. When elevated, PAI-1, an endothelial plasminogen activator inhibitor, is a risk factor for thrombosis and atherosclerosis [23]. Fibronectin plays a major role in cell adhesion, growth, migration, and differentiation, and it is important for processes such as wound healing and embryonic development. Altered fibronectin expression, degradation, and organization have been associated with several pathologies, including cancer, arthritis, and fibrosis [24,25]. MCP-1 is known as a chemokine for monocytes, neutrophils, lymphocytes, and macrophage recruitment. It has been shown that MCP-1 promotes macrophage-mediated tubular injury [26]. In the present study, we observed that the mRNA levels of most of the above-described proinflammatory and fibrotic genes in the kidneys of DOCS normotensive mice were upregulated, including IP-10, MCP-1, TGF-*β*, TNF-*α*, osteopontin (OPN), p65 (NF-kB), ICAM, PAI-1, fibronectin, and collagen type I and III. Furthermore, dietary K^+^ supplementation partially but significantly downregulated these genes (Figure 4 and Figure 5, Table 1). The preliminary results suggest that both potassium and sodium may modulate some proinflammatory and fibrotic genes for developing chronic kidney lesions independent of blood pressure in DOCS one-renin gene mice.

Potassium depletion (intracellular K^+^ concentration < 90 mmol) can increase the NALP3 inflammasome, which activates proinflammatory cytokines such as interleukin (IL)-1*β* and IL-18 [27], the latter of which is associated with an increased inflammatory infiltrate and severe kidney lesions [28]. In two-kidney, one-clip (2K1C) renovascular hypertensive one-renin gene mice, we have observed significant upregulation of RNA levels of Nlrp3, IL-1*β*, and IL-1*α* in renal artery-clipped kidneys with renal ischemia. Using a monoclonal antibody specific for IL1*β*, 200 µg intraperitoneally weekly for 6 weeks in 2K1C and sham mice, the treatment prevented the rise of PRA and BP in 2K1C mice and had no effect on PRA and BP in sham animals, suggesting that IL1 may participate in the regulation of renin secretion and the development of renin-dependent hypertension in the renin-dependent 2K1C renovascular mouse model [29].

Nevertheless, the mediators of progressive chronic kidney disease remain largely unsolved, especially the mechanisms leading to progressive renal inflammation and fibrosis. The inflammation and fibrosis in the kidney are generally preceded by glomerular and tubulo-interstitial infiltration by inflammatory cells. It is likely that the infiltrating inflammatory cells activate NF-kB, leading to the production and release of proinflammatory cytokines and adhesion molecules (e.g., IL-1*β*, ICAM) as well as profibrotic cytokines (e.g., TGF-*β*). The consequence is renal inflammation and fibrosis, leading to loss of function.

It is noted that the limitation of the present study is that inflammatory protein levels of the proinflammatory and fibrotic genes were not analyzed. In addition, the immunomodulatory cytokines IL-1, IL-18, etc. should be further investigated in the DOCS one-renin gene mouse model. Those need to be further studied and confirmed in the future.

## 4. Materials and Methods

### 4.1. Mice and Experimental Protocol

Experiments were performed on seven-week-old, male, wild-type back cross N_5–6_(129Ola/C57BL/6J) one-renin gene mice (weight 23 to 25 g) procured from the Institute of Pharmacology, University of Lausanne in Switzerland, that were homozygous for the Ren-1c gene locus. The back cross is necessary because a 129Ola mouse is a two-renin gene mouse strain characterized by hypertension (MBP: 148 ± 4 mmHg, HR: 564 ± 19 beats/min, BW: 30 ± 1.9 g, *n* = 5, unpublished data) and high plasma renin activity and concentration (PRA and PRC).

The experiments were approved by Consumer Affairs and Veterinary Affairs Department, Canton of Vaud, Switzerland (1571.1). All animals were housed in a room lighted 12 h/day at an ambient temperature of 22 ± 1 °C. The DOCS mouse model has been described earlier [7,8]; briefly, laboratory-made medical silicone implants filled with DOCA powder (Sigma; DOCA release rate: 21.3 ± 0.22 μg/h) were subcutaneously administered at the belly in uni-nephrectomies mice. Meanwhile, these mice received a 1% NaCl drinking solution. Five weeks later, some DOCS-treated mice started with a mixed drinking fluid of 1% NaCl and 0.4% KCl, which is considered the potassium-supplementation group (DOCS+KCl). Uni-nephrectomized mice without DOCA implants but receiving tap drinking water are considered the tap water group (TAP). All mice were kept on a regular pellet containing 2.2 mg Na^+^ and 8.5 mg K^+^/g (KLIBA NAFAG, Food ref. No.: 3200, Provimi Kliba AG CH-4303). The amount of 1% NaCl drink fluid intake from the sealed graduated bottle was ~30 mL/day in DOCS mice and about 5 times more than that in TAP water mice. Na^+^ and K^+^ intake in DOCS mice was ~112 mg and ~22.5 mg/day, respectively, including the amount of Na^+^ and K^+^ derived from the food (~3 g pellet/day). The dietary sodium-potassium ratio (Na/K) is ~8.5 using the mmol. In DOCS+KCl-treated mice, the amount of drink fluid and Na^+^ intake were the same as the DOCS mice, respectively, whereas K^+^ intake was 80 mg/day. Dietary Na/K was 2.4. Three hours (8:30–11:30) of mouse urine were collected from each mouse penile urethra by a suitable plastic tube without urine leakage in a custom-made small rodent animal urine collection station. Intra-arterial blood pressure and heart rate were measured in conscious mice. Blood and kidney were sampled by the end of 5, 8, and 11 weeks after the initial operation for biochemistry, molecular, and pathological examinations. Each group of mice consisted of 9–12 individuals.

### 4.2. Blood Pressure, Serum and Urinary Electrolytes, Creatinine, and Protein

Blood pressure (BP) and heart rate (HR) were recorded intra-arterially using a silicone/PE10 catheter and computerized data-acquisition system at a sampling rate of 500 Hz (Notocord Systems SA, Croissy, France) [8]. For the placement of an intra-arterial catheter, the mouse was anesthetized by inhalation of 1 to 1.5% isoflurane mixed with oxygen (1.5 L/min). The right carotid artery was exposed for a length of approximately 4 mm. A silicone/PE10 catheter filled with 0.9% NaCl solution containing heparin (300 IU/mL) was inserted into the artery. After ligation, the catheter was subcutaneously tunneled to exit at the back of the neck and fixed with a piece of scotch and dental cement. The mouse was allowed 3 to 4 h to recover from anesthesia and then placed into a Plexiglas tube for partial restriction of its movements. Thirty minutes later, the mouse catheter was connected to a pressure transducer, and BP and HR were then monitored every 20 s for 15 to 30 min. After BP and HR recording, 500 μL of blood was drawn from the carotid artery into 0.6-mL Multivette tubes containing gel/clot activator (Sarstedt). After blood sampling, the mouse was killed, and the kidney was rapidly excised, washed with cold PBS, dried with soft facial tissue, weighed, and cut into two parts. The half kidney was fixed in 3.8% formol and embedded in paraffin. Another half kidney was frozen in nitrogen and stored at −80 °C. To determine renal hypertrophy, the kidney weight index was calculated by the kidney weight (mg) to body weight (g) ratio (KW/BW, mg/g).

Urinary and serum Na and K concentrations were measured by flame photometry (IL-943; Instrumentation Laboratory, Milan, Italy). Creatinine concentration was assessed by the picric acid method (Cobas-Mira; Roche, Basel, Switzerland). Urinary proteins were quantified using a modified Lowry technique (BioRad DC protein assay, Bio-Rad Laboratories, Hercules, CA, USA). Urinary albumin values were divided by urine creatinine.

### 4.3. Renal Pathology

Kidney tissues were stained with PAS reagent. The glomerular injury was measured by qualitative scoring. A score of 0 out of 3 indicates no injury, 1 mild injury in less than a quarter of the glomerular tuft, 2 damage to more than a quarter of the glomerular tuft, and 3 damage to the whole glomerulus. Planimetric examinations of the glomerular cross-sectional area were performed as described. Renal fibrosis was judged to have no (score 0 = 0), little (score 1 = 1–10%), light (score 2 = 11–20%), moderate (score 3 = 21–40%), severe (score 4 = 41–60%), or severe fibrosis (score 5 = ≥ 61%). Sections post-fixed with 1% osmium tetroxide and embedded in araldite were observed under an electron microscope. Ultrathin sections were stained and photographed using a transmission electron microscope (EM 902; Carl Zeiss AG, Oberkochen, Germany).

### 4.4. Renal Proinflammatory Marker Genes

RNA Isolation and Quantitative RT-PCR: expression of proinflammatory marker genes was quantitatively assessed to examine the potential molecular mediators of mineralocorticoid/salt-induced renal damage. Kidneys were homogenized in TriPure Isolation Reagent (Roche Diagnostics Corp, Basel, Switzerland) using a Kinematica Polytron blender (Kinematica AG, Malters, Switzerland), and total RNA was extracted according to the kit protocol. Briefly, total RNA was treated for 30 min in the presence of DNase I (a DNA-free kit; Ambion Inc., Austin, TX, USA) and reverse-transcribed using the ImProm-2 Reverse Transcription System (Promega, Madison, WI, USA). Quantitative PCR was performed using the SYBR^®^ Premix ExTaq™ (Takara, Dalian, China) in a Lightcycler Instrument (Roche Diagnostics, Rotkreuz, Switzerland). Negative controls included amplification of distilled water and RNA samples that had not been reverse transcribed. The analysis of the data was performed using the 3.5 version of the Light Cycler software (Roche Diagnostics, Rotkreuz, Switzerland) [30]. cDNAs were amplified using specific mouse primers for qPCR (Table 3).

### 4.5. Statistical Analysis

All results are presented as mean ± SEM. Statistical comparisons between groups were performed by a 1-way ANOVA, followed by a Newman-Keuls test. *p* < 0.05 was considered the minimal level of significance. Statistical analyses were performed with the GraphPad PRISM 7

## 5. Conclusions

Taken together, the preliminary results of the present study provide new evidence that both potassium and sodium may modulate proinflammatory and fibrotic genes, leading to chronic renal lesions independent of blood pressure. Dietary potassium supplementation can partially reduce high mineralocorticoid and high sodium intake-induced chronic renal lesions, independent of blood pressure. The new finding may provide the opportunity to study the mechanism of chronic kidney diseases in patients with high circuiting aldosterone and high sodium consumption. Finally, a low sodium and potassium-enriched diet may have cost-effective benefits for the prevention of cardiovascular events and deaths in the adult population under a high sodium and low potassium diet [31,32,33].

## Figures and Tables

**Table 1:** Physiological parameters in control and DOCA/salt mice without and with potassium supplementation.

	TAP			DOCS			DOCS + KCl	
	5 Weeks	8 Weeks	11 Weeks	5 Weeks	8 Weeks	11 Weeks	8 + 3 Weeks	11 + 6 Weeks
Number of mice	9	12	12	10	9	10	11	12
Body weight (g)	27 ± 0.4	28 ± 0.4	28 ± 0.3	28 ± 0.5	29 ± 0.3	29 ± 0.4	29 ± 0.4	29 ± 0.4
MBP (mmHg)	113 ± 2	114 ± 2	114 ± 2	112 ± 2	110 ± 2	109 ± 3	109 ± 3	112 ± 3
Heart rate (beats/min)	604 ± 20	601 ± 12	591 ± 15	600 ± 6	585 ± 8	578 ± 12	596 ± 10	577 ± 12
Serum Na^+^ (mmol/L)	150 ± 1	151 ± 1	152 ± 1	153 ± 1	154 ± 1	154 ± 1	154 ± 1	154 ± 1
Kidney weight (mg)	204 ± 4	236 ± 9	253 ± 6	357 ± 8 ***	376 ± 9 ***	399 ± 12 ***	350 ± 6 ^†^	350 ± 4 ^††^
U_Na/Creat_	57 ± 4	54 ± 5	73 ± 6	289 ± 56 ***	366 ± 5 ***	389 ± 44 ***	250 ± 44	444 ± 86
U_K/Creat_	50 ± 2	55 ± 4	62 ± 4	56 ± 6	68 ± 3	62 ± 4	141 ± 14 ^†††^	204 ± 29 ^††^
U_Prot/Creat_ (g/mmol)	0.8 ± 0.1	0.7 ± 0.03	0.7 ± 0.1	1.7 ± 0.2 **	1.1 ± 0.1 **	1.1 ± 0.1 **	0.8 ± 0.1	0.5 ± 0.1 ^†^

MBP: mean blood pressure; TAP: tap water group; DOCS: DOCA/salt group; DOCS + KCl: DOCA/salt + KCl group; U_Prot/Creat_: urinary protein to creatinine ratio. Values are mean ± SEM; ** *p* < 0.01, *** *p* < 0.001 DOCS vs. TAP; ^†^ *p* < 0.05, ^††^ *p* < 0.01, ^†††^ *p* < 0.001 DOCS + KCl vs. DOCS.

**Table 2:** Renal vascular wall thickness and tubular hypertrophy in control and DOCS mice without and with potassium supplementation.

	TAP		DOCS		DOCS+KCl	
	8 weeks	11 weeks	8 Weeks	11 Weeks	8 + 3 Weeks	11 + 6 Weeks
Number of mice	9	10	11	8	9	12
Vascular wall thickness	none	none	yes	yes	focal	focal
Tubular hypertrophy	none	none	focal	yes	focal or no	focal or yes

**Table 3:** List of primers used for qPCR.

Gene	Sense Primer (5′-3′)	Antisense Primer (5′-3′)
PAI-1	GACTGGGTGGAAAGGCATAC	GCGTGTCAGCTCGTCTACAG
Fibronectin	TCCTGCCTGGGACAGAATAC	TGAATGAGTTGGCGGTGATA
Collagen type I	GATGGATTCCCGTTCGAGTA	AGGCCTCGGTGGACATTAG
Collagen type III	TTGGAATTGCAGGGCTAACT	AGGACCACGTTCCCCATTAT
IP-10	CCCACGTGTTGAGATCATTG	CACTGGGTAAAGGGGAGTGA
MCP-1	CCCACTCACCTGCTGCTACT	GCTGCTGGTGATCCTCTTGTA
TGF-β	CTGCTGACCCCCACTGATAC	GCTGAATCGAAAGCCCTGTA
TNF-α	CGTCAGCCGATTTGCTATCT	CGGACTCCGCAAAGTCTAAG
Osteopontin	TGCACCCAGATCCTATAGCC	CTCCATCGTCATCATCATCG
P65	GAGCCCATGGAGTTCCAGTA	TCGGGTAGGCACAGCAATAC
ICAM	GGGGAACCCATCTCCTAAGA	AGGCATGGCACACGTATGTA
ANP	ATGCTGGCAGCTAGGAGACA	AGGCCAAGACGAGGAAGAAG
α-skeletal actin	GGACCTGTACGCCAACAACG	AGCCACCGATCCACACTGAG
18S	CTCAACACGGGAAACCTCAC	AGACAAATCGCTCCACCAAC

## Data Availability

Data is unavailable due to privacy or ethical restrictions in the hospital. But it could be required via email.
